# Applications of cell free protein synthesis in protein design

**DOI:** 10.1002/pro.5148

**Published:** 2024-08-24

**Authors:** Ella Lucille Thornton, Sarah Maria Paterson, Michael J. Stam, Christopher W. Wood, Nadanai Laohakunakorn, Lynne Regan

**Affiliations:** ^1^ Centre for Engineering Biology, Institute of Quantitative Biology, Biochemistry and Biotechnology, School of Biological Sciences University of Edinburgh Edinburgh UK

**Keywords:** cell‐free protein synthesis, medium throughput, protein design, screening, surface immobilization

## Abstract

In protein design, the ultimate test of success is that the designs function as desired. Here, we discuss the utility of cell free protein synthesis (CFPS) as a rapid, convenient and versatile method to screen for activity. We champion the use of CFPS in screening potential designs. Compared to in vivo protein screening, a wider range of different activities can be evaluated using CFPS, and the scale on which it can easily be used—screening tens to hundreds of designed proteins—is ideally suited to current needs. Protein design using physics‐based strategies tended to have a relatively low success rate, compared with current machine‐learning based methods. Screening steps (such as yeast display) were often used to identify proteins that displayed the desired activity from many designs that were highly ranked computationally. We also describe how CFPS is well‐suited to identify the reasons designs fail, which may include problems with transcription, translation, and solubility, in addition to not achieving the desired structure and function.

## 1 INTRODUCTION

Protein design is not 100% successful. Current machine‐learning approaches have dramatically increased the probability that a designed protein folds and functions as intended (Notin et al., 2024). Not only are functional protein mutants more feasible to design, but also entirely de novo proteins (Ferruz et al., 2022; Watson et al., 2023). Nevertheless, making and characterizing the designed proteins is still the ultimate test. The challenge now is to identify (from the order of 10 to 100 top ranked designs) those which function as desired. Moreover, it is important to delineate why designs fail: to distinguish between those which are problematic with regard to expression or solubility and those which do not adopt the correct functional structure (Huang et al., 2016).

Cell free protein synthesis (CFPS) provides a convenient approach both for identifying functional designs, and for understanding the reasons designs do not behave as desired (Jin & Hong, 2018). In CFPS, the DNA encoding the desired protein is incubated with a cell extract, appropriately supplemented with amino acids, tRNA etc.—the majority of examples discussed here use *E. coli* extracts (Gregorio et al., 2019; Perez et al., 2016). CFPS using extracts from other prokaryotic and eukaryotic organisms is also possible, with a variety of associated advantages and disadvantages (Batista et al., 2021; Moore et al., 2021; Tinafar et al., 2019).

Alternatively, a fully defined system, known as PURE (Shimizu et al., 2001), can be used, in which the minimal components required for protein synthesis are purified and combined. To date, PURE has only been applied using *E. coli*‐derived translation machinery, and it typically results in substantially lower yields of the desired protein than *E. coli* extract‐based CFPS (Garamella et al., 2016; Lavickova & Maerkl, 2019).

CFPS offers many advantages in comparison to the production of recombinant proteins in live microbes (Laohakunakorn et al., 2020). First, because protein synthesis is decoupled from cellular growth, the constraint of host cell viability is removed, permitting the production of cytotoxic proteins. Second, because all genomic DNA is removed, the reaction is programmed solely with the DNA of interest, either a plasmid or a linear PCR product (Niederholtmeyer et al., 2015; Sun et al., 2014). Third, because the reactions are open, conditions such as ionic strength, pH, temperature, and redox potential can be tailored for the production of specific proteins (Michel & Wüthrich, 2012). Fourth, extremely small volumes are used, making the protocol well‐suited for a screening step, whilst keeping costs low.

There are, however, challenges associated with the use of CFPS. Perhaps the most significant is establishing the protocols in a laboratory that is familiar with other methods of protein production. We maintain that it is worth overcoming this ‘activation barrier’ because there are many advantages to using CFPS for screening on the scale currently required. To address this issue, the CFPS community are actively engaged in the development of more robust and reproducible protocols (Dopp et al., 2019).

One limitation that could be an issue for the production of longer proteins by CFPS is incomplete translation. This has been estimated as a ‘processivity loss per codon’ of about 10^−3^, corresponding to only about one third of proteins synthesized being full length after 1 KB of translation (Doerr et al., 2021; Hurst et al., 2017; Sin et al., 2016). Interestingly, premature truncation seems to be less of a problem in eukaryotic CFPS, potentially due a lower frequency of translational pausing (Ramachandiran et al., 2000).

In this review we focus on two aspects of the applicability of CFPS to protein design. First, as a convenient way to make and test the function of designed proteins. Second, as a way to explore why designs fail—delineating transcription, translation, aggregation or folding problems. The number of different designs that can reasonably be screened manually in an open CFPS system is of the order of hundreds. The use of 96 or 384 well plates or microfluidic devices is typical. Reaction volumes are commensurately small. It is this ‘medium throughput’ scale that has proven most accessible in the context of screening designed proteins. Using CFPS as a platform to not only test for desired protein activity, but also assess why designs have failed provides a uniquely rich data set, which can then be fed back into a model to inform the next round of designs. Therefore, CFPS is a simple, informative and accessible technique to use in the protein design and validation pipeline.

**FIGURE 1:**
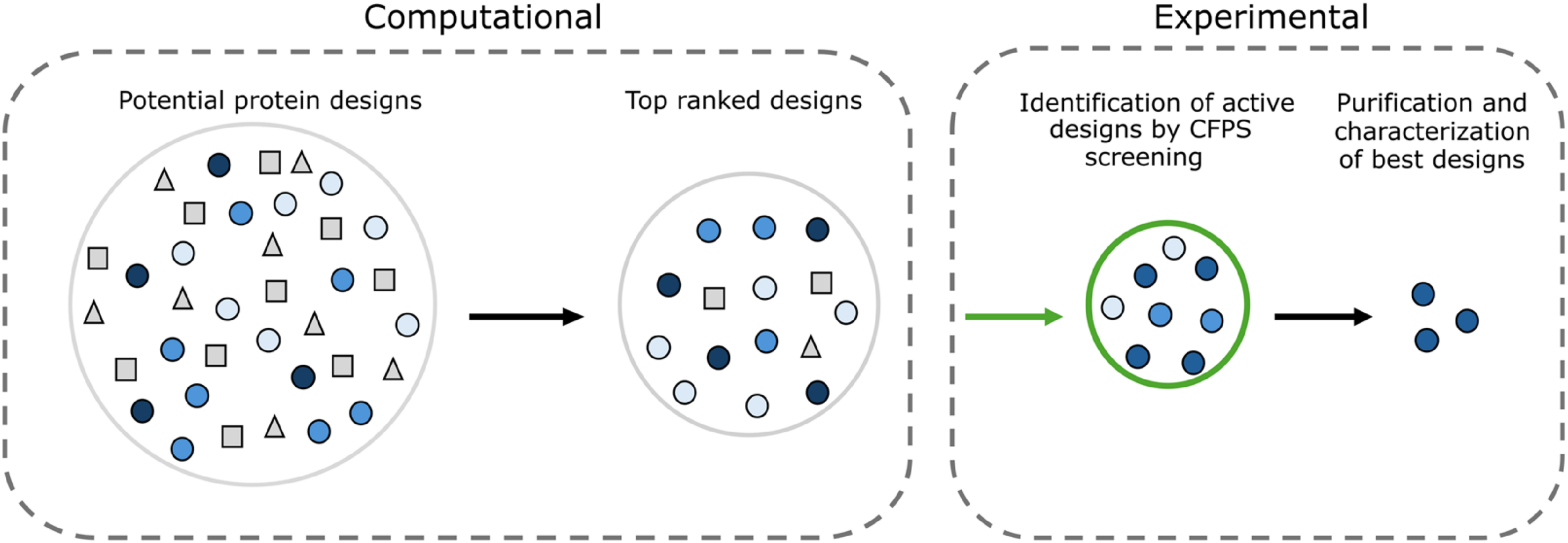
Schematic representing the identification of functional protein designs. A large number of potential protein designs are created computationally. These designs are ranked and the top designs identified. These computationally top‐ranked designs are tested in CFPS—for production and activity. The proteins that display the highest activity in CFPS are then studied further, for example by purification and biophysical characterization.

The strategy often followed is: design thousands of sequences; rank them by desired property; choose ten to a hundred designs to express in CFPS; determine the activity of CFPS produced proteins and identify the best. CFPS is well suited for this purpose as the open nature (i.e., not within cells) allows for straightforward screening of desired function directly from solution (Laohakunakorn, 2020). Based on the results of CFPS characterization, it is typical to choose a few exemplar proteins to express at a larger scale in bacteria, purify and characterize. The rationale for standard purification after initial CFPS screening, is that much larger quantities of protein can be produced—suitable for more detailed characterisations, including structure determination. Larger amounts of material also allow the specific activity of designed proteins to be readily determined (Figure 1).

## 2 SCREENING FOR ACTIVITY

The focus of this review is medium throughput screening methods and their use in combination with modern computational protein design.

There are many examples, beyond the scope of this review, where CFPS has been applied in a high throughput format. For example, mutants of the membrane protein α‐hemolysin were produced by CFPS, incorporated into liposomes, and pore‐forming activity identified using high throughput microfluidic sorting (Fujii et al., 2013). In another, encapsulation of cell‐free reactions allowed for genotype–phenotype linkage and directed evolution of more active protease mutants (Holstein et al., 2021).

A recent report described a fully automated method to explore the thermal stability/activity landscape of a protein, using robotics combined with CFPS to screen thousands of enzymes (Rapp et al., 2024). Mutant enzymes were made by CFPS and screened for enhanced thermal tolerance by incubation at various temperatures before screening for retained enzymatic activity via a fluorescence‐based assay.

**FIGURE 2:**
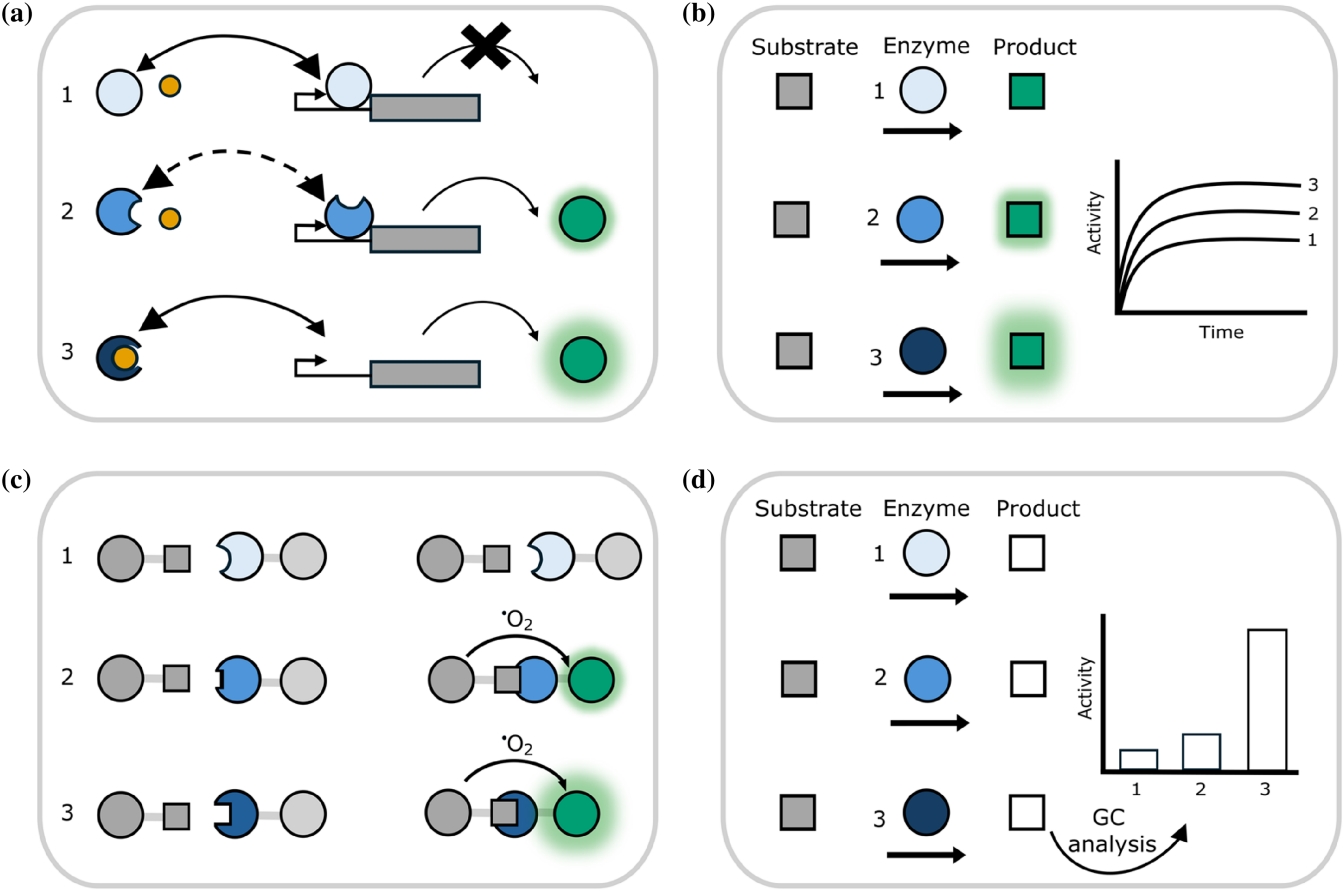
Examples of protein functionality that can be assessed using CFPS. In each example, we show in cartoon form how three different designed proteins would behave in the assay. We represent more functional proteins by darker shades of blue. (a) Designing a transcriptional repressor to bind to a small molecule inducer. In this case, designed proteins are screened for their ability to bind the inducer (yellow). Proteins that function as desired are identified by an increase in GFP production in response to the presence of the small molecule. (b) Identifying designed proteins with increased enzymatic activity via production of a colored or fluorescent product. (c) Screening for binding. Protein variants that bind to another molecule (gray square) are identified via proximity induced fluorescence of the beads to which the protein and molecule are attached, in an AlphaLISA® assay. Other methods of detection, such as FRET, could also be used. (d) In some cases, detection of enzyme activity is only possible by removing and analyzing a sample from the CFPS, such as by gas chromatography (GC).

By combining CFPS screening for function with computational protein design, the sequence space to be assessed can be narrowed significantly. Many characterisations can be performed within the CFPS reaction plate or microfluidic device (Figure 2a–c). However, sometimes there is no convenient in situ fluorescence or colorimetric assay, and samples must be taken, for example for mass spectroscopy or gas chromatography analysis of the products of an enzymatic reaction (Figure 2d). Occasionally, purification of the protein product is required before further characterisations can be performed. These different methods to screen protein designs are simplified by the fact CFPS is an open system, and does not require a lysis step after expression, which would reduce throughput. Each of these approaches is exemplified by case studies in the following paragraphs.

One design campaign sought to create a vanillin biosensor by designing variants of the repressor, qacR, with DNA binding activity modulated by binding vanillin (rather than its natural inducer; de los Santos et al., 2016). Over 10^10^ computational designs were ranked and reduced to just 10 variants to be screened, followed by testing of another 17 candidates—two of which displayed the desired properties. Screening in CFPS used a two‐plasmid system, where successful binding of vanillin by a re‐designed repressor resulted in expression of GFP. This allowed for fast screening of many mutants in parallel, leading to easy identification of successful mutants by increased green fluorescence (Figure 2a). These experiments illustrate an additional point. The authors first tested this system in *E. coli* cells, but found it was not functional. They hypothesized the problem was the impermeability of the cell wall to vanillin. The system worked well in CFPS, demonstrating the advantage of an open system.

A recent study reported the use of CFPS to screen azoreductases (Rolf et al., 2022), characterizing 10 enzymes (5 of which were previously uncharacterised) for activity against several different substrates and co‐substrates. By using PURE CFPS rather than lysate‐based CFPS to synthesize the enzymes, the activity of *E. coli's* endogenous azoreductase was removed, thus decreasing the background. Further, the authors demonstrated how CFPS conditions could be optimized to increase soluble protein yield: the addition of chaperone proteins, and reduction of temperature for the CFPS reaction from 37°C to 20°C vastly improved the amount of soluble, active enzyme produced. The essence of the screen was to use CFPS to produce the enzyme, followed by a microplate‐based colorimetric assay to monitor changes in the concentration of substrates over time (Figure 2b).

CFPS has been applied as a screening platform for antibody discovery, specifically in the later‐stages of functional characterization of the binding characteristics of around 100 different antibodies (in this case, Covid‐19 neutralizing antibodies; Hunt et al., 2023). These antibodies had already been identified by traditional methods, but CFPS allowed for detailed characterization of their binding specificity. In this example, CFPS enabled ready manipulation of the solution conditions to favor disulfide bond formation, in a fashion that would not have been feasible within *E. coli* (Figure 2c).

Although a colorimetric or fluorescence‐based assay to readout activity is the most straightforward to implement, other assays can also be used to test samples taken directly following CFPS. For example, mutants of Old Yellow Enzyme were produced in CFPS and screened for their capability to reduce different substrates, assaying the activity via gas chromatography detection of products (Quertinmont & Lutz, 2016; Figure 2d).

For some designed proteins it is desirable to synthesize them by CFPS, but then to purify using an affinity tag before performing activity assays. For example, Madani and colleagues used large‐language models to generate thousands of designs for lysozyme from five different structural classes (Madani et al., 2023). They tested 100, across all five structural classes, using CFPS to produce the enzymes at small scale. CFPS protein production was followed by purification and assessment of enzymatic activity. About 80% of the enzymes could be expressed in CFPS, and about 70% of the proteins produced by CFPS showed some activity. Interestingly, when they tried *E. coli* expression and purification of five of these proteins, one was not expressed, two were insoluble, and two expressed in soluble form, but because of their enzymatic activity they resulted in lysis of the *E. coli* cells in which they were expressed. These results illustrate how CFPS allows for assessment of designs that might fail due to insolubility in the more concentrated environment of an *E. coli* cell, or that might kill the cell if successfully expressed.

## 3 SURFACE CAPTURE OF CFPS PRODUCED PROTEIN

Surface capture of the protein product is highly desirable when assessing protein function (Meldal & Schoffelen, 2016). By capturing the protein product to the solid support of the surface, it can be separated from the CFPS reagents through washing of the surface. This allows for testing the effects of different conditions on protein activity, such as pH, temperature, buffer components, and reactivity towards different substrates—with the ability to test each mutant under multiple conditions by simply washing the surface and replacing the reaction buffer.

**FIGURE 3:**
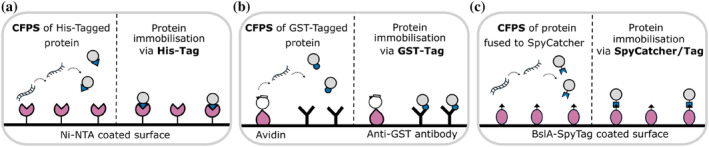
Examples of surface capture strategies from CFPS. (a) A His‐tagged protein produced by CFPS is captured to a Ni‐NTA coated surface. (b) Biotinylated DNA is bound to an avidin coated surface. CFPS from this DNA produces the protein product fused to GST (glutathione S‐transferase), which can then be captured to the surface by anti‐GST antibodies. (c) BslA‐SpyTag forms a self‐assembling protein monolayer. SpyCatcher‐protein fusion made by CFPS is captured via a covalent bond to the SpyTag of the BslA‐SpyTag surface coating.

An early demonstration of cell‐free expression and in situ capture to a surface was the development of PISA (Protein In Situ Array; He, 2001). PISA achieves surface capture of the expressed protein of interest (which is fused to a His‐tag) using a nickel coated surface. The principal has been extended to include also the surface attachment of the DNA being transcribed (via biotin in the DNA binding to an avidin coated surface; Ramachandran et al., 2004), and has been adapted to a microfluidic setting (Geertz et al., 2012). Recently, a self‐assembling protein surface has been used to covalently capture cell‐free synthesized proteins to the surface (Thornton et al., 2022; Figure 3).

## 4 CFPS TO IDENTIFY THE REASONS WHY A DESIGN FAILED

**FIGURE 4:**
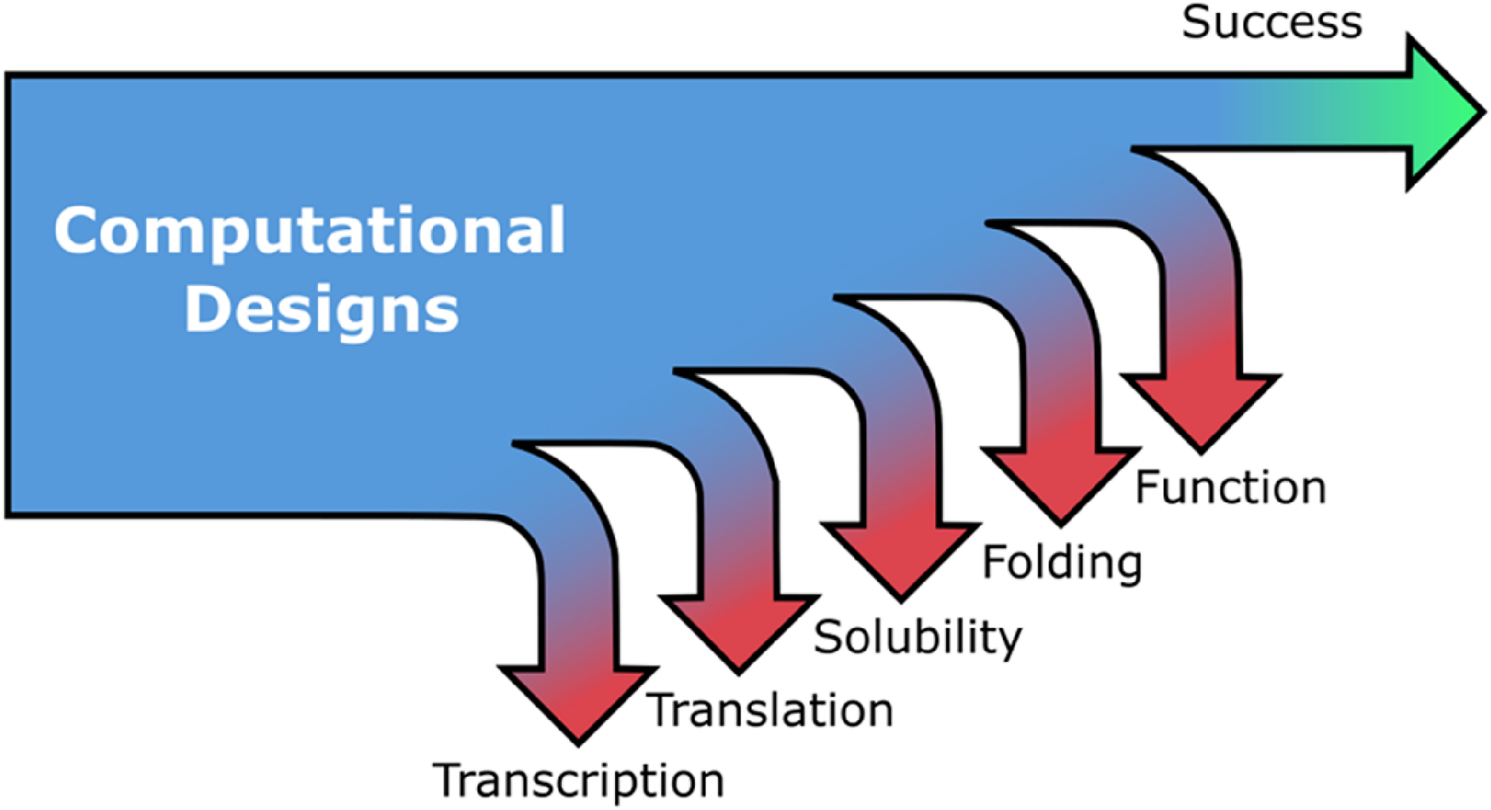
Schematic illustration of the different points at which a protein design may fail. The point of failure can be identified using CFPS.

A key issue with current protein design is that designs may ‘fail’ for many reasons, in addition to the design not achieving the desired structure and function (Huang et al., 2016). CFPS allows the cause of protein design failures—including issues with transcription, translation, aggregation and misfolding, to be identified (Figure 4; Dopp et al., 2019; Katzen et al., 2005; Kwon & Jewett, 2015; Silverman et al., 2019; Swank et al., 2019; Tsuboyama et al., 2023).

Transcription can be tested by adding a dye‐binding aptamer sequence to the end of the mRNA that encodes the protein of interest (Ouellet, 2016). Successful translation can be tested by a few different techniques. The most straightforward method would be to fuse a fluorescent protein to the end of the protein of interest (POI), however this is quite a bulky addition to the POI and therefore using fluorescence as a proxy for translation might underestimate how much of the POI has been successfully made. A recent study uses a tetra‐cysteine mini helix as a tag to the POI, which in combination with a FlAsH dye, indicates protein concentration via fluorescence (Willi et al., 2024). Another approach is to incorporate radioactive amino acids into TCA (trichloroacetic acid) precipitable proteins (Coleman et al., 2004).

Solubility can be tested either by incorporation of a fluorescent lysine residue or a radioactive amino acid into the protein of interest. The partitioning of the fluorescence or radioactivity between pellet and supernatant can then be determined (Coleman et al., 2004).

Protein folding in CFPS has recently been examined by a high‐throughput proteolysis assay, allowing for the fast assessment of protein folding of 100,000's of protein designs (Tsuboyama et al., 2023).

Using approaches such as these, experimental data can be collected to distinguish between different reasons for design failures. Subsequently data‐driven methods can be used to identify the properties of designed proteins that may lead to failure and the results of such analyses incorporated into future protein design algorithms.

## 5 SUMMARY

In summary, CFPS is well‐suited to be incorporated as an intermediary screen in many different protein design campaigns. Although establishing it anew in a laboratory may not be appealing, the benefits over traditional methods of expression are significant. The wide range of different functional assays that can be implemented in a cell‐free system also enables the function (rather than just structure) of different designs to be readily assessed. Moreover, researchers in the CFPS field are continually improving the scope and robustness of the method. The application of machine‐learning methods to the challenges of protein design has resulted in a spectacular increase in the frequency of successful designs (Sumida et al., 2024; Yeh et al., 2023). Using more traditional ‘physics based’ or ‘physics plus statistics based’ approaches, often it would be necessary to screen many potential designs to identify the one(s) with the desired activity. For example, screening designs for desired binding properties could include a yeast display screen, with the capacity to screen around 5 × 10^5^ different variants (Fleishman et al., 2011).

Current machine‐learning based approaches can achieve success rates up to 10 to 15% (Kortemme, 2024). Thus, far fewer designs need to be screened in order to identify the successful one(s). At the medium throughput level, CFPS provides an accessible technique for researchers to test many protein designs for functionality in parallel. One can thus anticipate CFPS becoming an even more significant tool to facilitate protein design in the future.

## AUTHOR CONTRIBUTIONS

**Ella Lucille Thornton:** Conceptualization; writing – original draft; writing – review and editing. **Sarah Maria Paterson:** Conceptualization; writing – original draft; writing – review and editing. **Michael J. Stam:** Conceptualization; writing – review and editing; writing – original draft. **Christopher W. Wood:** Conceptualization; writing – review and editing; writing – original draft. **Nadanai Laohakunakorn:** Conceptualization; writing – original draft; writing – review and editing. **Lynne Regan:** Conceptualization; writing – original draft; writing – review and editing.

## CONFLICT OF INTEREST STATEMENT

The authors declare that this review article was written in the absence of any commercial or financial relationships that could be construed as a potential conflict of interest.
